# Treatment of Dysentery with Creasote

**Published:** 1875-01

**Authors:** Jno. R. Cushing

**Affiliations:** Harrison County, Texas


ARTICLE II.

### ON THE TREATMENT OF DYSENTERY WITH CREASOTE.

BY JNO. R. CUSHING, M.D., HARRISON COUNTY, TEXAS.

An intractable epidemic "bloody flux," which prevailed in this vicinity in the summer and fall of 1873, led me to experimenting with the creasote. Its well known property of allaying the irritable stomach of our autumnal fevers impressed me that it would be valuable in the treatment of the disease, when opiates and other astringents proved nugatory or of little effect. Its *modus operandi* I will not attempt to discuss at this time, whether it acts as an astringent or an alterative, but I can say it acted admirably in my hands in its curative power over the disease. Whether it was a peculiar phase of the disease or not, I cannot say, as it will require experience in future epidemics to decide. But my faith is strong in its remedial power, and will continue so until proved otherwise.

The attacks of the disease were in most cases sudden, without chill or diarrhœa. The bowels were generally constipated, and tender over the sigmoid flexure and colon. The discharges were pure blood and mucus, with little tormina, but excessive tenesmus. The opiate plan of treatment had a controling but not a curative influence upon the disease, for it invariably terminated (when the case did not die) in chronic ulceration of the lower rectum, frequently producing spasm of the sphincter so intolerable that the patient could not avoid screaming upon every evacuation.

Upon adopting the creasote, in combination with opiates, I was happily surprised beyond all expectation. It accomplished everything and did it in a few days, as well as obviating that ugly *after symptom*, so distressing in my preceding cases. In obstinate diarrhœas I find the preparation equally as good as in the dysentery.

When ulceration of the rectum remained after the subsidence of the disease, I found the acetate plumbi, in combination with tinct. opii, used as enemas, all that was necessary ; what was very strange, enemas of creasote did but very little good.

The following cases from my note book will illustrate my plan of treatment :

Case 1. A lady, ætat.26 ; sick two days before called in ; had been taking teas and laudanum. Found bowels costive, with great tormina and tenesmus. Sulph. magnesia ℥i, tinct. opii ʒi, water ℥vi. Tablespoonful every thirty minutes until it acted. Cold cloths to the perineum, and cloths saturated with the liniment, aq. ammon. ℥ss, oil sassafras ℥ss, tinct. opii ʒi, tinct. arnica ʒi, ol. olive and kerosene *aa* ℥ij, kept constantly applied to the bowels. After the action of the salts, commenced the following formula : ℞. Creasote, gtt xx ; Acetic Acid, gtt xl ; Morphia, gr. ij ; Aqua, ℥ij. M. Teaspoonful every two hours until relieved. Well in three days.

Case 2. Young man, 18 years ; taken about six hours previous to my seeing him ; put him immediately upon the creasote mix. with the liniment ; cured in thirty-six hours.

Case 3. Gentleman, aged about 50 years ; had been sick about four days ; had been taking opium pills, with acet. plumbi ; considerable tenesmus, with dejections of bloody mucus ; great prostration ; bowels tympanitic. Prescribed creasote mix. with turpentine stupes to the bowels ; whisky ℥i every three hours. In fifty-six hours the bowels were controlled. Creasote mix. every four hours; whisky, toddy and egg nog, pro re nata. Discharged cured on the fourteenth day.

Case 4. Lady, aged about 20. Cured on third day. Creasote mix. only used.

Cases 5, 6, 7 and 8, in one family, all down with hemorrhage of bowels; no tormina or tenesmus; great tenderness over the bowels, and irritable stomach. Creasote mix., turpentine stupes, and stimulants. All cured and discharged between seventh and fourteenth day. These cases simulated peritonitis, yet I am satisfied it was not that disease, for there was no inflammatory fever or other serious indications of that disease. The disease was evidently subacute inflammation of the lower bowels, with ulceration of the hemorrhoidal veins. My treatment proved efficacious in almost every instance. The simplicity of the treatment and the rapidity of cure, compared with the remedies I had previously used, demonstrated that creasote was an important adjuvant, in fact, the main one, in combatting the disease. I should have some hesitation in prescribing the remedy in acute disease accompanied with high febrile excitement.

In all diarrhœas that I meet with, my main reliance is in the creasote. In the treatment of the disease in children I substitute tr. opii camp. instead of the morphia, and lessen the dose according to age.

For the ulcerations in the rectum when they occurred, I used enemas of ulmus fulva and the acet. plumbi with tr. opii—½ ʒ plumbi and gtt xxv tr. opii to about ℥ ij of menstruum—every three, four or six hours, as the case required.

